# Diminished 25‐OH vitamin D_3_ levels and vitamin D receptor variants are associated with susceptibility to type 2 diabetes with coronary artery diseases

**DOI:** 10.1002/jcla.23137

**Published:** 2019-12-03

**Authors:** Lei Ma, Shujin Wang, Heming Chen, Lin Cui, Xiaoxiang Liu, Hua Yang, Guohong Li, Songfang Liu, Ting Qi, Hongyan Tian

**Affiliations:** ^1^ Department of Endocrinology Ninth Hospital of Xi'an Xi'an China; ^2^ Department of Peripheral Vascular Disease The First Affiliated Hospital of Xi'an Jiaotong University Xi'an China; ^3^ Department of Endocrinology Ankang Central Hospital Ankang China; ^4^ Department of Nephrology Ninth Hospital of Xi'an Xi'an China; ^5^ Departments of Cardiology Shaanxi Provincial People's Hospital Xi'an China

**Keywords:** Chinese, coronary artery disease; vitamin D, polymorphism, Type‐2 diabetes, vitamin D receptor

## Abstract

**Background:**

Role of plasma vitamin D and genetic variants of its receptor (VDR) in susceptibility to different diseases has been documented. Various studies in different populations have been highlighted strong associations with diabetes and cardiovascular diseases. Vitamin D deficiency has been linked with the development of type 2 diabetes (T2D) and the onset of coronary artery diseases (CAD). However, the role of vitamin D in predisposition to CAD in patients with T2D is ill‐defined.

**Materials and Methods:**

We enrolled 674 Chinese T2D patients, and based on clinical phenotype, patients were further categorized into patients with (n = 138) or without coronary artery disease (n = 536). Five hundred twenty‐one healthy subjects from similar geographical areas, free from diabetic or coronary disorders, were enrolled as controls. Serum levels of 25‐OH vitamin D were quantified by ELISA. Common VDR (*FokI*, *TaqI*, *BsmI,* and *ApaI*) polymorphisms were genotyped by polymerase chain reaction‐restriction fragment length polymorphism (PCR‐RFLP).

**Results:**

Patients with T2D displayed lower levels of 25‐OH vitamin D compared with healthy controls. Furthermore, T2D patients with CAD clinical phenotype had the lowest levels of vitamin D. Prevalence of *FokI* and *TaqI* mutants was significantly higher in diabetic patients when compared to controls. Interestingly, Tt genotype was more frequent in the artery disease group in comparison with T2D patients without heart involvement. Combined analysis of VDR polymorphisms and serum levels of vitamin D revealed a significant role in predisposition to T2D with or without CAD.

**Conclusions:**

Lower vitamin D levels and variants of VDR polymorphisms (*FokI* and *TaqI*) are associated with susceptibility to T2D and clinical manifestation.

## 1 INTRODUCTION

Diabetes is considered an important health‐related complication in humans that results when the pancreas cannot produce a sufficient amount of insulin (Type 1 diabetes) or when the body cannot effectively utilize the insulin which it makes (Type 2 diabetes) (T2D).^1^ It is estimated that by 2030, around 552 million people around the world will be diagnosed with T2D indicating the growing gravity of this health problem.^2^ Despite such disease burden associated with socioeconomic issues, the pathogenesis of T2D is not yet completely understood. The most vital physiological feature of T2D is insulin resistance, which is characterized by the impaired response to insulin in insulin‐sensitive tissues, and β‐cell failure resulting in β‐cell dysfunction and reduced β‐cell mass.^3^

Diabetes needs dedicated management by patients as well as health care professionals. An important clinical practice for diabetes is the aggressive management of risk factors to prevent complications that may lead to enhanced mortality. Among several such risk factors, a major one causing mortality in T2D subjects is cardiovascular disease (CVD). CVD in patients with T2D is often found to be more severe and complex and that leads to a higher rate of complications compared to those with non‐diabetic phenotypes.^4^ About one‐fourth of total mortality in patients with T2D has been contributed to CVD clinical phenotype.^5^ A multinational study including patients from twenty‐eight countries revealed a prevalence of CVD in patients with T2D ranged from 21.6% to 34.2%.^6^ Among different CVDs, the incidence of coronary artery diseases (CAD) increases along with age and occurs in younger age population with diabetes.^7^, ^8^ In patients with T2D, CAD possesses complexities characterized by small, diffuse, calcified multi‐vessel disease.^9^ An estimate showed about 75% of subjects with T2D die as a result of related heart diseases including CAD.^10^

The human body synthesizes vitamin D with exposure to ultraviolet (UV) B rays, and the importance of vitamin D to maintain good health has been demonstrated elegantly. Vitamin D deficiency is widely prevalent across the globe, and the problem is more severe in elderly patients.^11^ Deficient levels of vitamin D are associated with susceptibility to a wide range of diseases, including T2D and CVD. About 70%‐90% of patients with T2D displayed deficient or insufficient plasma levels of vitamin D and believed to be associated with different clinical phenotypes of T2D.^12^, ^13^, ^14^ An observational study including a more substantial number of women demonstrated an increased risk of T2D in those with lower levels of vitamin D.^15^ Diminished vitamin D levels have been linked with impaired glycemic controls.^16^, ^17^ Supplementation of vitamin D in patients with T2D has shown promising findings by lowering blood cholesterols 18 and HDL levels.^19^ There is growing evidence suggesting an association of lower vitamin D levels with susceptibility to CAD and mortality.^20^, ^21^ A recent report highlighted connotation between deficient vitamin D levels and severity of CAD.^22^

In addition to vitamin D levels, vitamin D receptors (VDR) polymorphisms are also associated with genetic susceptibility to T2D and CAD patients. Vitamin D is believed to exert its effect through VDR, and genetic variations in the VDR gene lead to dysfunctional vitamin D signaling. Several studies have been executed in different populations to decipher the role of VDR polymorphisms in T2D^23^, ^24^, ^25^, ^26^, ^27^, ^28^, ^29^, ^30^, ^31^, ^32^, ^33^, ^34^, ^35^, ^36^, ^37^, ^38^, ^39^and CAD.^40^, ^41^, ^42^, ^43^, ^44^, ^45^ To the best of our knowledge, studies on the importance of vitamin D or VDR polymorphisms in T2D patients with CAD are lacking. In the present study, we enrolled patients with T2D from the Chinese cohort and investigated the importance of vitamin D and VDR in patients with T2D concerning CAD.

## 2 MATERIALS AND METHODS

### 2.1 Study subjects

In the present study, a total of 674 subjects with T2D, visiting outpatient department (OPD) or admitted at the department of peripheral vascular disease, the first affiliated hospital of Xi'an Jiaotong University during the period of March 2015 to May 2019, were enrolled. Patients were considered to have confirmed CAD if they had experienced prior episodes of ST‐elevation myocardial infarction or proven to have CAD angiographically. Age‐ and sex‐matched healthy subjects hailing from a similar geographical area without a history of any heart‐related anomalies or history of diabetes were enrolled as controls. Participants were divided into three groups, such as healthy controls (Group 1, n = 521), subjects with T2D without CAD (Group 2, n = 536), and subjects with T2D with CAD (Group 3, n = 138). The staging for diabetes was performed on the basis of fasting blood sugar (FBS), postprandial blood sugar (PPBS), and glycosylated hemoglobin (HbA1c) levels according to American Diabetic Association^46^ (FBS > 150 mg/dL, PPBS > 200 mg/dL, HbA1c > 7 mmol/mol). For all patients, standard care was followed for anti‐diabetic treatment such as appropriate diet, oral hypoglycemic drugs with or without insulin, and only insulin treatment. Therapeutic intervention for CAD included multifactorial treatment such as the use of statins (95%), aspirin (86%), and blockade of the renin‐angiotensin‐aldosterone system (98%).

Different biochemical parameters such as triglycerides, total cholesterol, LDL, and HDL of patients and healthy controls were measured in the laboratory facility available in the hospital. Patients were categorized into three different groups based on vitamin D levels as deficient (0‐20 ng/mL), insufficient (20‐30 ng/mL), and sufficient (>30 ng/mL) according to guidelines of the Institute of Medicine and endocrine society.^47^ Informed consent was collected from each participant, and the study protocol was approved by the Institutional Human Ethical Committee of the first affiliated hospital of Xi'an Jiaotong University.

### 2.2 Collection of serum

About 2 mL of blood (without anticoagulant) was collected intravenously from each patient immediately after the enrollment of patients. Blood was also collected from healthy controls. Serum was separated after centrifuging blood at 2000rpm for 5 minutes and was stored at −20°C till further use.

### 2.3 Determination of serum vitamin 25‐OH vitamin D levels

Total vitamin D [25(OH)D] in sera was estimated using enzyme‐linked immunosorbent assay (ELISA) kit (R&D Systems) according to the manufacturer's instructions.

### 2.4 Isolation of whole genomic DNA

About 200 microliters of whole blood were used for isolation of genomic DNA from patients with T2D and healthy controls. The genomic DNA was isolated by QIAamp DNA mini kit (Qiagen) according to manufactures directions.

### 2.5 Genotyping of VDR polymorphisms

Common polymorphisms in vitamin D receptor (VDR) were genotyped by polymerase chain reaction followed by restriction fragment length polymorphism (PCR‐RFLP), as described earlier.^48^ Briefly, four sets of primers were employed for amplification of DNA fragment containing respective polymorphic site as follows (*FokI*: F‐5′‐GCA CTG ACT CTG GCT CTG AC‐3′ and R‐5′‐ACC CTC CTG CTC CTG TGG CT‐3′; *TaqI*: F‐5′‐TCC TGT GCC TTC TTC TCT ATC‐3′ and R‐5′‐CTA GCT TCT GGA TCA TCT TGG‐3′; *BsmI*: F‐5′‐GGA GAC ACA GAT AAG GAA ATA C‐3′ and R‐5′‐CCG CAA GAA ACC TCA AAT AAC A‐3′, *ApaI*: F‐5′‐TGG GCA CGG GGA TAG AGA AG‐3′ and R‐5′‐ACG GAG AAG TCA CTG GAG GG‐3′) in different reaction mixture. For each PCR, 1 unit of Taq polymerase, 0.5 µL of 10 nm deoxynucleotide triphosphate mixture, and 2 µL of genomic DNA from patients or controls were used. The PCR cycle was denaturation for 95°C for 5 minutes followed by 35 cycles of denaturation at 95°C for 30 seconds followed annealing time of 45 seconds at 60°C and at extension at 72°C for 45 seconds. The final extension was performed at 72°C for 10 minutes. Amplicon size for different reaction products was as follows (*FokI*: 341bp, *TaqI*: 172bp, *BsmI*: 248bp, and *ApaI*: 177bp). Amplicons were digested with a respective restriction enzyme and analyzed for genotypes of subjects. Forty amplicons were randomly selected and subjected to sequencing for confirmation of genotyping methods. We observed a 100% concordant reading between these two methods.

### 2.6 Statistical analysis

Genotype and allele frequencies in different clinical categories of patients and healthy controls were quantified by direct counting. Prevalence of VDR polymorphisms in healthy controls and T2D patients with or without CAD was compared by Fisher's exact test. *P* value, odds ratio, and 95% confidence interval were calculated. *P* value <.01 was taken a significant after Bonferroni correction for four single nucleotide polymorphisms (0.05/4 = 0.01). Serum levels of 25‐OH vitamin D_3_ in different clinical categories were compared by analysis of variance followed by Tukey's post‐test. Distributions of vitamin d status among patients with T2D and healthy controls were compared by Fisher's exact test. All statistical tests were performed by GraphPad Prism 7.04. Deviation of VDR genotypes distributions from Hardy‐Weinberg equilibrium (HWE) in the studied population, if any, was evaluated by in house formulated Microsoft Excel sheet.

## 3 RESULTS

### 3.1 Baseline characteristics

The baseline characteristics for patients and healthy controls are shown in Table 1. Out of 674 subjects diagnosed with T2D, 138 patients (20.4%) had CAD. The average age of participants in the three study groups was comparable. No gender disparity among three study groups was noticed. As shown in Table 1, levels of FBS, PPBS, HbA1C (%), systolic blood pressure, diastolic blood pressure, and triglycerides were found to be significantly higher in diabetic subjects when compared to healthy controls. Although no difference was observed in total cholesterol and LDL levels among three study groups, diabetic patients had significantly lower levels of HDL as compared to healthy controls. Additionally, the prevalence of hypertension in patients with T2D was more frequent when compared to healthy controls.

**Table 1:** Baseline characteristics in patients and controls

Parameters	Healthy controls	Patients with T2D (without CAD)	Patients with T2D (with CAD)
Total subjects	521	536	138
Age (y)	62.4 ± 3.3	63.6 ± 1.2	60.3 ± 2.3
Sex			
Male	246 (47.2%)	257 (47.9%)	62 (44.9%)
Female	275 (52.8%)	279 (52.1%)	76 (55.1%)
Fasting plasma glucose (mg/dl)	81.5 ± 9.4	137.7 ± 12.4	139.7 ± 14.2
Postprandial blood sugar (mg/dl)	137.2 ± 15.7	208.4 ± 16.3	211.8 ± 19.5
HbA1C (%)	4.6 ± 0.8	7.7 ± 1.6	7.9 ± 1.9
Systolic blood pressure (mmHg)	119.5 ± 11.9	129.8 ± 11.7	130 ± 10.9
Diastolic blood pressure (mmHg)	82.9 ± 7.7	88 ± 11.2	90 ± 11.6
Total cholesterol (mg/dl)	163.5 ± 23.7	159.9 ± 21.9	162.3 ± 28.4
HDL cholesterol (mg/dl)	54.3 ± 14.6	48.7 ± 12.4	46.7 ± 16.6
LDL cholesterol (mg/dl)	131.2 ± 16.8	132.6 ± 15.3	135.5 ± 15.6
Triglycerides (mg/dl)	131.3 ± 34.2	140.4 ± 29.7	147.6 ± 31.9
Duration of diabetes (y)	NA	8.8 ± 1.1	9.3 ± 0.7
Hypertension	173 (33.2%)	429 (80.03%)	119 (86.2%)
Alcohol intake	299 (57.3%)	317 (59.1%)	77 (55.7%)
Smoking habits			
Never	369 (70.8%)	389 (72.5%)	99 (71.7%)
Current	152 (30.2%)	147 (27.5%)	39 (28.2%)
Anti‐diabetic treatment	NA		
Diet		105 (19.6%)	25 (18.3%)
OHA		376 (70.1%)	96 (69.6%)
OHA + Insulin		27 (5.0%)	9 (6.7%)
Only insulin		28 (5.3%)	8 (5.4%)

Furthermore, we compared all biochemical parameters among T2D patients with or without CAD and observed significantly higher triglyceride levels in T2D patients with CAD compared to T2D patients without CAD phenotype. Furthermore, hypertension is more frequent in T2D patients with CAD than those without CAD. However, other biochemical parameters remained comparable among these two clinical groups (T2D patients with or without CAD).

### 3.2 Serum levels of vitamin D in different clinical categories of enrolled subjects

**Figure 1:**
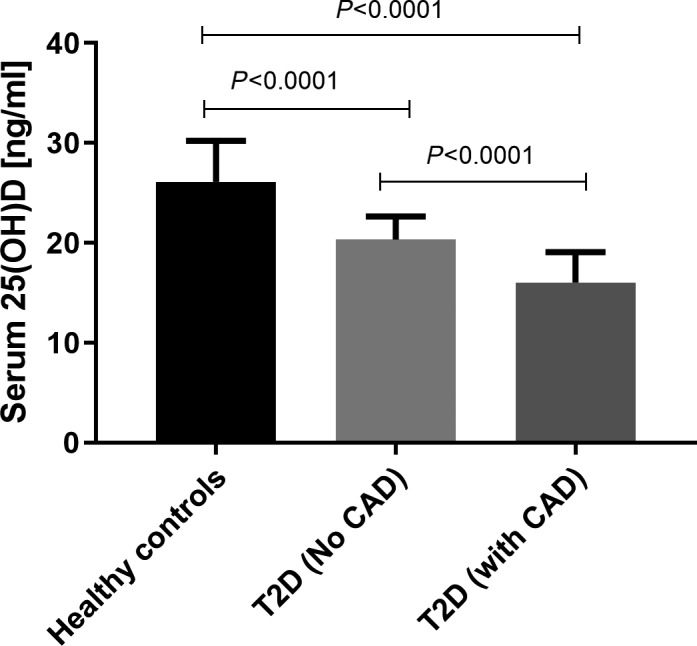
Serum 25‐OH vitamin D levels in T2D patients and healthy controls. Serum levels of 25‐OH vitamin D were quantified by ELISA, and mean levels of vitamin D were compared among three clinical categories, healthy controls, T2D patients with CAD, and T2D patients without CAD by ANOVA followed by Tukey's post‐test. *P* < .05 was considered statistically significant

25‐OH vitamin D_3_ levels were measured in the serum of subjects belonged to the three study groups, and results are shown in Figure 1. Levels were found to be 26.13 ± 0.18 ng/mL, 20.33 ± 0.1 ng/mL, and 16.03 ± 0.26 ng/mL, respectively, in healthy controls, T2D patients without CAD and with CAD. Irrespective of clinical phenotype, patients with T2D had a lower level of 25‐OH vitamin D when compared to healthy controls with minimal level observed in T2D patients with CAD. Interestingly, T2D subjects with CAD manifestation had a significantly lower level of vitamin D compared with diabetic subjects without CAD (*P* < .0001).

Furthermore, based on earlier reports,^49^, ^50^ all subjects were grouped into three different categories (a) deficient (<20 ng/mL), (b) insufficient (20‐30 ng/mL), and sufficient (>30 ng/mL) and their distributions were investigated in healthy controls and different clinical categories of patients with T2D. As shown in Table 2, the majority of healthy individuals in the studied population had insufficient vitamin D levels (64.7%). Prevalence of subjects with insufficient and deficient vitamin D levels was significantly higher in T2D patients with (insufficient: *P* < .0001, OR = 5.23; deficient: *P* < .0001, OR = 25.36) or without CAD (insufficient: *P* < .0001, OR = 2.70; deficient: *P* < .0001, OR = 6.84) when compared to healthy controls. It is important that the percentage of patients with deficient serum vitamin D was significantly higher in T2D with CAD clinical phenotype than those without CAD (*P* = .001, OR = 3.70).

**Table 2:** Plasma levels of vitamin D in healthy controls and different clinical categories of T2D patients

Vitamin D levels	HC (n = 521)	T2D without CAD (n = 536)	T2D with CAD (n = 138)	HC vs T2D without CAD; *P* value, OR, 95% CI	HC vs T2D with CAD; *P* value, OR, 95% CI	T2D without CAD vs with CAD; *P* value, OR, 95% CI
Sufficient (>30 ng/mL)	162 (31.1%)	71 (13.3%)	9 (6.5%)	Ref, 1	Ref, 1	Ref, 1
Insufficient (20‐30 ng/mL)	337 (64.7%)	399 (74.4%)	98 (71.0%)	**<.0001, 2.70, 1.98 to 3.67**	**<.0001, 5.23, 2.68 to 10.33**	.08, 1.93, 0.94 to 3.94
Deficient (<20 ng/mL)	22 (4.2%)	66 (12.3%)	31 (22.5%)	**<.0001, 6.84, 3.89 to 11.8**	**<.0001, 25.36, 10.68 to 56.65**	**.001, 3.70, 1.61 to 8.64**

Note

Data are shown as numbers (%). Prevalence of vitamin D among different clinical groups was compared by Fisher's exact test.

Statisticaly significant values are shown in bold.

### 3.3 Distribution of VDR polymorphisms in the healthy Chinese population

Common genetic polymorphism in the VDR gene (*FokI*, *TaqI*, *BsmI,* and *ApaI*) was genotyped by PCR‐RFLP, and a total of 521 healthy Chinese controls were typed in the present investigation and data are shown in Table 3. For *FokI*, *TaqI,* and *BsmI* polymorphisms, the wild type (FF:66%; TT: 64%; BB: 85%) was more frequent in the studied healthy group compared with heterozygous (Ff: 31%; Tt: 33%; Bb: 12%) and homozygous mutant (ff: 3%; tt: 3%; bb: 3%). However, heterozygous for *ApaI* polymorphism (Aa: 43%) was most prevalent when compared to wild type (AA: 39%) and homozygous mutant (aa: 18%). Distribution of genotypes for *FokI* (*χ* ^2^ = 0.29; *P* = .58) and *TaqI* (*χ* ^2^ = 1.21; *P* = .26) was in Hardy‐Weinberg equilibrium (HWE) and other two VDR polymorphisms deviated from the equilibrium condition (*BsmI*: *χ* ^2^ = 34.19; *P* < .001, *ApaI*: *χ* ^2^ = 5.28; *P* = .02). Deviation of genotype distribution from HWE could be due to several factors such as genotyping errors, population stratification, or selection pressure.^51^ The patients and controls enrolled in the present study were from an Asian ethnic group, and we have adopted the robust technique for genotyping of samples. As the prevalence of the certain infectious disease is high in the Chinese population and such disease are believed to play a major role in natural selection,^52^ these infectious diseases could be a possible reason for HWE deviation.

**Table 3:** Distribution of VDR polymorphisms in healthy controls and type 2 diabetes patients with and without CAD

VDR Polymorphisms	Genotype or Allele	HC (n = 521)	T2D patients without CAD (n = 536)	T2D patient with CAD (n = 138)	HC vs T2D without CAD		HC vs T2D with CAD		T2D without CAD vs T2D with CAD	
					*P* value	OR (95% CI)	*P* value	OR (95% CI)	*P* value	OR (95% CI)
*FokI* (rs2228570)	Genotype									
	FF	344 (66)	193 (36)	44 (32)	1	Ref	1	Ref	1	Ref
	Ff	161 (31)	306 (57)	88 (64)	**<.0001**	**3.38 (2.61 to 4.38)**	**<.0001**	**4.27 (2.81 to 6.43)**	.26	1.26 (0.83 to 1.88)
	ff	16 (3)	37 (7)	6 (4)	**<.0001**	**4.12 (2.29 to 7.59)**	.03	2.93 (1.10 to 7.97)	.66	0.71 (0.29 to 1.73)
	Allele									
	F	849 (81)	692 (65)	176 (64)	1	Ref	1	Ref	1	Ref
	f	193 (19)	380 (35)	100 (36)	**<.0001**	**2.41 (1.97 to 2.95)**	**<.0001**	**2.49 (0.93 to 3.35)**	.83	1.03 (0.78 to 1.35)
*TaqI* (rs731236)	Genotype									
	TT	333 (64)	230 (43)	39 (28)	1	Ref	1	Ref	1	Ref
	Tt	172 (33)	278 (52)	94 (68)	**<.0001**	**2.34 (1.81 to 3.01)**	**<.0001**	**4.66 (3.10 to 7.01)**	**.001**	**1.99 (1.31 to 2.97)**
	tt	16 (3)	28 (5)	5 (4)	**<.004**	**2.53 (0.67 to 4.84)**	.07	2.66 (1.02 to 7.14)	1	1.05 (0.42 to 2.72)
	Allele									
	T	838 (80)	738 (69)	172 (62)	1	Ref	1	Ref	1	Ref
	t	204 (20)	334 (31)	104 (38)	**<.0001**	**1.85 (0.76 to 2.27)**	**<.0001**	**2.48 (1.86 to 3.31)**	.04	1.33 (1.01 to 1.75)
*BsmI* (rs1544410)	Genotype									
	BB	443 (85)	418 (78)	115 (83)	1	Ref	1	Ref	1	Ref
	Bb	63 (12)	91 (17)	19 (14)	.01	1.53 (1.08 to 2.15)	.56	1.16 (0.67 to 1.99)	.36	0.75 (0.45 to 1.29)
	bb	15 (3)	27 (5)	4 (3)	.05	1.90 (1.03 to 3.60)	1	1.02 (0.36 to 2.91)	.36	0.53 (0.19 to 1.43)
	Allele									
	B	949 (91)	927 (86)	249 (90)	1	Ref	1	Ref	1	Ref
	b	93 (9)	145 (14)	27 (10)	**.0009**	**1.59 (1.21 to 2.10)**	.63	1.10 (0.70 to 1.74)	.10	0.69 (0.44 to 1.07)
*ApaI* (rs7975232)	Genotype									
	AA	203 (39)	225 (42)	58 (42)	1	Ref	1	Ref	1	Ref
	Aa	224 (43)	228 (42)	63 (46)	.54	0.91 (0.70 to 1.19)	1	0.98 (0.65 to 1.48)	.75	1.07 (0.71 to 1.60)
	aa	94 (18)	83 (16)	17 (12)	.21	0.79 (0.55 to 1.13)	.15	0.63 (0.35 to 1.13)	.55	0.79 (0.44 to 1.43)
	Allele									
	A	630 (60)	678 (63)	179 (65)	1	Ref	1	Ref	1	Ref
	a	412 (40)	394 (37)	97 (15)	.19	0.88 (0.74 to 1.05)	.18	0.82 (0.62 to 1.08)	.67	0.93 (0.70 to 1.22)

Note

Data are shown as numbers (%). Prevalence of VDR polymorphisms among different clinical groups was compared by Fisher's exact test.

Statisticaly significant values are shown in bold.

### 3.4 Variants of VDR polymorphisms are associated with susceptibility to T2D and/or clinical severity

We investigated the possible association of VDR polymorphisms (*FokI*, *TaqI*, *BsmI,* and *ApaI*) with susceptibility/ resistance against the development of type 2 diabetes. As shown in Table 3, prevalence of heterozygous mutant (Ff) and minor allele (f) for *FokI* polymorphism was significantly higher in T2D patients with (Ff: *P* < .0001, OR = 4.27; f: *P* < .0001, OR = 2.49) or without CAD (Ff: *P* < .0001, OR = 3.38; f: *P* < .0001, OR = 2.41) compared with healthy controls. Similar observation was also noticed for *TaqI* polymorphism: “Tt” genotype and “t” were significantly high in T2D patients [with CAD (Ff: *P* < .0001, OR = 4.66; f: *P* < .0001, OR = 2.48), without CAD (Ff: *P* < .0001, OR = 2.34; f: *P* < .0001, OR = 1.85)] than controls. Interestingly, homozygous mutant for *FokI* (ff) and *TaqI* (tt) polymorphisms were also more frequent in T2D patients without CAD when compared to healthy controls (ff: *P* < .0001, OR = 4.12; tt: *P* = .004, OR = 2.53). The genotype distribution of *BsmI* and *ApaI* polymorphism was comparable among T2D patients with or without CAD and controls. However, the minor allele of VDR *BsmI* polymorphism was significantly higher in T2D patients without CAD phenotype than controls (*P* = .0009, OR = 1.59). Most interestingly, patients with T2D harboring heterozygous genotype for VDR *TaqI* polymorphism (Tt) had a higher chance of developing CAD clinical manifestation (*P* = .001; OR = 1.99) indicating an essential role of *TaqI* polymorphism on the determination of clinical severity in Chinese T2D patients (Table 3).

### 3.5 Association of Combined VDR polymorphisms and serum 25‐OH vitamin D with T2D

Vitamin D shows its effect through vitamin D receptor (VDR). Variants in the VDR gene lead to lower levels of VDR proteins or deformed receptors, which would lead to lowered signaling process. As we observed the significant association of VDR *FokI* and *TaqI* variants with susceptibility to T2D development and diminished levels of serum levels of 25‐OH vitamin D_3_ in diabetic patients with or without CAD clinical phenotype, we hypothesized that combined VDR polymorphisms and serum levels analysis would be linked with development of T2D and responsible for different clinical manifestations. In the present study, we considered *FokI* and *TaqI* polymorphisms for combined analysis since we observed their independent association with a predisposition to T2D. As shown in Table 4, combined *FokI*/vitamin D levels (FF/deficient, Ff/deficient, ff/deficient, Ff/insufficient, ff/insufficient) were more frequent in T2D patients compared with healthy controls. These combinations of VDR polymorphism and vitamin D levels are believed to be associated with diminished signaling. In contrast, the prevalence of FF/insufficient and Ff/sufficient was significantly higher in controls compared with patients. Interestingly, T2D patients with Ff/vitamin deficient combination were predisposed to T2D patients with CAD.

**Table 4:** Distribution of *FokI* and *TaqI* polymorphisms and vitamin D status in healthy controls and T2D patients with or without CAD

VDR polymorphism/ vitamin D status	HC (n = 521)	T2D patients without CAD (n = 536)	T2D patient with CAD (n = 138)	HC vs T2D without CAD		HC vs T2D with CAD		T2D without CAD vs T2D with CAD	
				*P* value	OR (95% CI)	*P* value	OR (95% CI)	*P* value	OR (95% CI)
*FokI* polymorphism/ Vitamin D status									
FF/ Sufficient	110 (21)	26 (5)	1 (0.7)	1	ref	1	ref	1	ref
Ff/ Sufficient	46 (9)	40 (7)	8 (6)	**<.0001**	**3.67 (2.02 to 6.62)**	**.0006**	19.13 (2.73 to 214)	.14	5.2 (0.81 to 59.7)
ff/ Sufficient	6 (1.1)	5 (1)	0	.05	3.52 (1.09 to 12.77)				
FF/ insufficient	224 (43)	138 (26)	33 (24)	**<.0001**	**2.60 (1.64 to 4.15)**	**<.0001**	16.21 (2.76 to 167.5)	.05	6.21 (1.06 to 65.96)
Ff/ insufficient	105 (20)	235 (44)	61 (44)	**<.0001**	**9.46 (5.85 to 15.17)**	**<.0001**	63.9 (11.28 to 652.2)	.03	6.74 (1.22 to 70.74)
ff/ insufficient	8 (1.53)	26 (5)	4 (3)	**<.0001**	**13.75 (5.43 to 32.73)**	**.0003**	55 (6.95 to 673)	.35	4 (0.57 to 50.56)
FF/ deficient	10 (2)	29 (5)	10 (7)	**<.0001**	**12.27 (5.37 to 28.22)**	**<.0001**	110 (14.15 to 1198)	.02	8.96 (1.27 to 100.7)
Ff/ deficient	10 (2)	31 (6)	19 (14)	**<.0001**	**13.12 (5.81 to 29.91)**	**<.0001**	209 (31.81 to 2186)	**.0008**	**15.94 (2.69 to 172)**
ff/ deficient	2 (0.3)	6 (1)	2 (1.3)	**.001**	**12.69 (2.90 to 63.08)**	**.002**	110 (7.93 to 1573)	.12	8.66 (0.82 to 128.9)
*TaqI* polymorphism/ vitamin D status									
TT/ Sufficient	103 (20)	32 (6)	1 (0.7)	1	ref	1	ref	1	ref
Tt/ sufficient	53 (10)	35 (6.5)	8 (6)	.01	2.12 (1.19 to 3.81)	**.001**	**15.55 (2.24 to 174)**	.06	7.31 (0.96 to 83.41)
tt/ sufficient	6 (1.1)	4 (0.7)	0	.26	2.14 (0.64 to 7.70)				
TT/ insufficient	220 (42)	165 (31)	26 (19)	**<.0001**	**2.41 (1.56 to 3.78)**	**<.0001**	**77.25 (14.44 to 782)**	.14	5.04 (0.86 to 53.54)
Tt/ insufficient	108 (21)	213 (40)	67 (49)	**<.0001**	**6.34 (3.99 to 9.86)**	**<.0001**	**63.9 (11.33 to 651.7)**	**.003**	**10.07 (1.67 to 104.7)**
tt/ insufficient	9 (1.7)	21 (4)	5 (4)	**<.0001**	**7.51 (3.01 to 17.22)**	**<.0001**	**57.22 (5.92 to 675.2)**	.07	7.61 (0.87 to 92.07)
TT/ deficient	10 (2)	33 (6)	12 (9)	**<.0001**	**10.62 (4.58 to 23.38)**	**<.0001**	**123.6 (16.93 to 1328)**	**.005**	**11.64 (1.82 to 128.2)**
Tt/ deficient	11 (2)	30 (5.5)	19 (14)	**<.0001**	**8.77 (3.84 to 18.3)**	**<.0001**	**177.9 (27.66 to 1863)**	**.0001**	**20.27 (2.97 to 217.4)**
tt/ deficient	1 (0.2)	3 (0.5)	0	.04	9.65 (1.37 to 126.3)				

Note

Data are shown as numbers (%). Prevalence of combined vitamin D status and VDR polymorphisms among different clinical groups were compared by Fisher's exact test.

Statisticaly significant values are shown in bold.

Similarly, VDR *TaqI* polymorphism and serum vitamin D_3_ combined analysis also revealed a significant association of Tt/insufficient, tt/insufficient, TT/deficient, and Tt/deficient combination with susceptibility to T2D. Interestingly, T2D patients with CAD had a higher prevalence of Tt/insufficient, TT/deficient, and Tt/deficient combination when compared to patients without CAD phenotype suggesting possible causal factors for the development of clinical severity in patients with T2D.

## 4 DISCUSSION

The present investigation aimed to examine the association of vitamin D level and its receptor variants with susceptibility of type 2 diabetic and clinical severity in the Chinese population. We observed diminished vitamin D levels in T2D patients with or without CAD when compared to healthy controls. Vitamin D‐deficient frequent in patients with T2D and interestingly highly prevalent in T2D patients with CAD clinical phenotype compared with diabetic patients without CAD. Furthermore, with subjects to coronary artery diseases. Furthermore, *TaqI* and *FokI* variants and minor alleles were significantly associated with predisposition to T2D. It is important that the combined analysis of vitamin D levels and VDR polymorphisms revealed their association with the development of T2D with or without CAD manifestations in the Chinese population.

Cholesterols are believed to play a significant role in heart‐related disorders and T2D. In the present study, no significant difference was marked in total cholesterol level and LDL among three study groups, the HDL level was found to be markedly lower in diabetic subjects as compared to healthy controls. However, such a difference was not noticed between T2D patients with or without CAD. Earlier epidemiological studies have demonstrated the association of lower HDL cholesterol with increased risk of type 2 diabetes^53^, ^54^ and our data were also in line with these observations. Furthermore, we observed higher levels of triglycerides in patients with T2D when compared with healthy controls corroborating with earlier findings^55^, ^56^ and triglycerides has been served as a potent marker of type 2 diabetes.^57^ Interestingly, the levels of triglyceride were significantly high in T2D patients with CAD compared with diabetic patients without CAD. Enhanced level of triglycerides has been considered as an independent predictor for CVD, and a fasting triglyceride level of more than 150mg/dl is mostly considered as an accepted criterion for defining individuals at high risk of CVD.^58^, ^59^ However, the role of triglycerides as a risk factor for developing CAD in type 2 diabetics is still unclear.^60^ Our data showing higher triglycerides level in T2D subjects with CAD indicating an important role of triglycerides in susceptibility to CAD and lowering of this molecule would possibly reduce the risk of heart diseases in T2D subjects.

Hypertension is a common clinical characteristic in patients with diabetes, and frequency depends on the duration of the disease, age of patients, sex, ethnicity, body mass index, and history of glycemic control.^61^ In the present study, we observed a higher prevalence of hypertension in patients with T2D in comparison with healthy controls. Interestingly, a higher frequency of hypertension was noticed in T2D patients with CAD (85.4%) when compared to diabetic patients without CAD (78.9%) indicating a possible role of hypertension in determining clinical complexity in T2D patients with reference to heart involvement. Moreover, no significant association of other risk factors such as FBS, PPBS, HbA1C, alcohol intake, and smoking habits between two patient groups was observed, indicating non‐involvement of these parameters in CAD pathogenesis in a studied Chinese cohort.

Lower levels of vitamin D have been associated with T2D compared with non‐diabetic controls.^62^, ^63^, ^64^, ^65^ In line with the earlier observations, we observed significantly lower levels of serum 25(OH) vitamin D in T2D subjects (both with and without CAD) as compared to healthy controls. Interestingly, T2D patients with CAD clinical conditions had the lowest levels of serum vitamin D in comparison with those without heart involvement. A direct association between hypovitaminosis D and cardiac complications has been adequately presented in many studies. Vitamin D deficiency is related to endothelial dysfunction and enhanced the risk of CVD.^66^, ^67^ The deficiency of this micronutrient has also been linked with cardiovascular events such as myocardial infarction, congestive heart failure, and sudden cardiac death.^66^, ^68^ Experimental evidence showed the presence of vitamin D receptors in the heart of rats and believed to play major role in intracellular calcium homeostasis.^69^ Furthermore, vitamin D deficiency leads to enhanced contractility, hypertrophy, and fibrosis in rats.^70^ The role of vitamin D in T2D patients with CAD remains conflicting. A report in Italian patients with T2D showed an inverse correlation between lower vitamin D levels and CVD in patients with T2D.^71^ In contrast, no significant association of vitamin D levels and severity of coronary atherosclerosis was observed in Polish patients.^72^ Our present data were showing significant hypovitaminosis D in T2D subjects with CAD indicating the possible role of the low vitamin in causing CAD in T2D subjects.

Biomolecules exert their effect through receptors. Deform or lower expression of receptors affects downstream signaling of biomolecules. VDR is a crucial molecule for vitamin D‐related signaling, and variations in the VDR gene have been attributed to hampered signaling and associated with a wide range of diseases. Four common VDR variants (*FokI*, *TaqI*, *BsmI,* and *ApaI*) have been investigated in genetic association studies and are believed to alter the signaling process. Several studies have been carried out in different populations for possible genetic association of VDR polymorphisms with susceptibility to T2D but yielded contradictory observations. A recent meta‐analysis including earlier published data revealed a significant association of *FokI* variants with susceptibility to T2D only with the Chinese population, and a weak association was noticed with *BsmI* variants.^37^ Similar results were also reported in another meta‐analysis but failed to demonstrate a possible link with other genetic variants (*ApaI*, *BsmI,* and *TaqI*).^33^

Further, independent genetic association studies in the Han Chinese population demonstrated a significant association of *BsmI* 35 and *FokI* 39 with T2D development. In the present study, we observed a significant association of *FokI* and *TaqI* variants with a predisposition to T2D. In line with our observations, patients with T2D from northeast India^32^ and Arab Emirates^73^ demonstrated a significant correlation with susceptibility to T2D. However, the association of *FokI* was not proved in Tunisian,^28^ Caucasian,^24^ Brazilian,^31^ and Egyptian^25^ patients with T2D. The mechanism of how VDR *FokI* and *TaqI* variants associated with T2D susceptibility is not known. Possibly subjects with VDR *FokI* and *TaqI* variants suppress vitamin D signaling and predisposed to the development of T2D.

Various studies have been conducted in different populations to understand the possible association of VDR polymorphisms with a predisposition to CAD. Although a recent meta‐analysis showed the absence of association between VDR polymorphisms (*FokI*, *TaqI*, *BsmI,* and *ApaI*) with susceptibility to CAD, reports from Iranian,^43^ Egyptian,^43^ and Chinese^41^ cohort showed a significant association of *FokI* variant with susceptibility to CAD. Our study was also corroborated with earlier observations: variants of *FokI* and *TaqI* were significantly higher in T2D patients with CAD phenotype. Interestingly, *TaqI* heterozygous mutant was significantly higher in T2D patients with CAD phenotype when compared to those without CAD. These observations altogether suggesting the importance of VDR polymorphisms on predisposition to CAD in patients with T2D.

Nowadays, combined analysis of vitamin D and VDR polymorphisms has been the most accepted form of association investigation on various diseases. In the present study, we observed heterozygotes or homozygous mutants of *FokI* and *TaqI* polymorphism and insufficient or deficient vitamin D levels with a predisposition to T2D development. It is important that combined genotype and serum vitamin D levels for *FokI* (Ff/deficient) and *TaqI* polymorphism (Tt/ insufficient, TT/deficient and Tt/deficient) were significantly more prevalent higher in T2D patients with CAD phenotype compared to those without CAD, indicating an important role of combined vitamin D and VDR variants with development of CAD in patients with T2D. Although the exact mechanism of how these genotypes phenotypes combination associated with CAD predisposition in patients with T2D is not known, possibly lower vitamin D and variants in VDR significantly altered vitamin D signaling process could lead to heart‐related deformities.

The present study has several limitations, and those need to be disclosed. Firstly, we have considered only four SNPs in the VDR gene, and other polymorphisms in the VDR gene are not included. Secondly, the number of samples included in the current investigation is limited. Thirdly, the mechanism of how VDR variants diminished vitamin D signaling is not investigated in this study.

In conclusion, lower levels of vitamin D and genetic variants of the VDR gene (*FokI* and *TaqI*) are associated with susceptibility to T2D and CAD. Furthermore, the combined analysis revealed a significant role of both serum vitamin D and VDR polymorphism on predisposition to patients with T2D or diabetic with CAD phenotype. However, more studies in other population including more samples are required for validation of our findings.
